# Targeting innovative therapeutic approaches to the hallmarks of aging to combat Alzheimer’s disease

**DOI:** 10.4103/NRR.NRR-D-25-00966

**Published:** 2025-12-30

**Authors:** Michal Izrael, Orli Miriam Frenkel

**Affiliations:** 1 Research & Development Department, Leverage Bio Ltd., Tel Aviv, Israel; 2 The Faculty of Biology, Technion Israel Institute of Technology, Haifa, Israel; 3 Hasharon Hospital, Rabin Medical Center, Petach Tikva, Israel

**Keywords:** aging hallmarks, Alzheimer’s disease, cellular senescence, genomic instability, neurodegeneration, proteostasis dysfunction, regenerative decline, rejuvenation factors, secretome

## Abstract

Aging is the leading risk factor for neurodegenerative diseases, including Alzheimer’s disease. Mounting evidence implicates twelve interconnected hallmarks of aging, such as genomic instability, mitochondrial dysfunction, cellular senescence, and altered intercellular communication, as core contributors to cognitive decline. In this review, we will first delineate the hallmarks of aging and their mechanistic roles according to their functions in the aging brain and Alzheimer’s disease. These hallmarks can be grouped into four major functional clusters: (i) Genomic and epigenomic instability, (ii) proteostasis and organelle dysfunction, (iii) cellular fate and regenerative decline, and (iv) cellular senescence. Then, we provide an overview of innovative therapeutic approaches aimed at modifying these hallmarks, focusing on the emerging paradigm of supplementation of rejuvenation factors that are derived from young plasma, stem cell secretomes, or their derivatives (e.g., extracellular vesicles). Finally, we discuss key aging-related biological factors that can influence Alzheimer’s disease progression and evaluate their potential as therapeutic targets.

## Alzheimer’s Disease and the Hallmarks of Aging: A Functional Framework

Alzheimer’s disease (AD) is a progressive neurodegenerative disorder and the leading cause of dementia worldwide, accounting for approximately 70% of cases (Breijyeh and Karaman, 2020). AD is clinically characterized by a gradual decline in memory, executive function, and behavior, ultimately leading to severe cognitive impairment and loss of independence. Globally, an estimated 57 million people are currently living with dementia, and with global aging, the burden of AD is projected to ~triple, reaching over 139 million cases by 2050 (Gustavsson et al., 2023). The socio-economic burden of AD is substantial. Worldwide costs of dementia care are estimated at over USD 2.8 trillion annually, encompassing medical treatment, long-term care, and productivity loss of caregivers (Nandi et al., 2022). Beyond financial costs, AD places immense emotional and social strain on patients, families, and healthcare systems. The pathology of AD includes extracellular amyloid-β (Aβ) plaques, intracellular neurofibrillary tangles composed of hyperphosphorylated tau, synaptic dysfunction, and neuroinflammation. Both genetic and sporadic factors contribute to disease onset: mutations in *APP*, presenilin-1 (*PSEN1*), and *PSEN2* cause rare familial AD, while the *APOE ε4* allele is the strongest genetic risk factor for late-onset AD. Despite decades of research, current therapies remain largely symptomatic, and no disease-modifying treatment is yet available (De Strooper and Karran, 2016; Long and Holtzman, 2019). Together, the epidemiological trends, devastating symptoms, and socio-economic burden of AD underscore the urgent need for innovative therapeutic strategies, including those targeting aging-related mechanisms.

**Figure 1:**
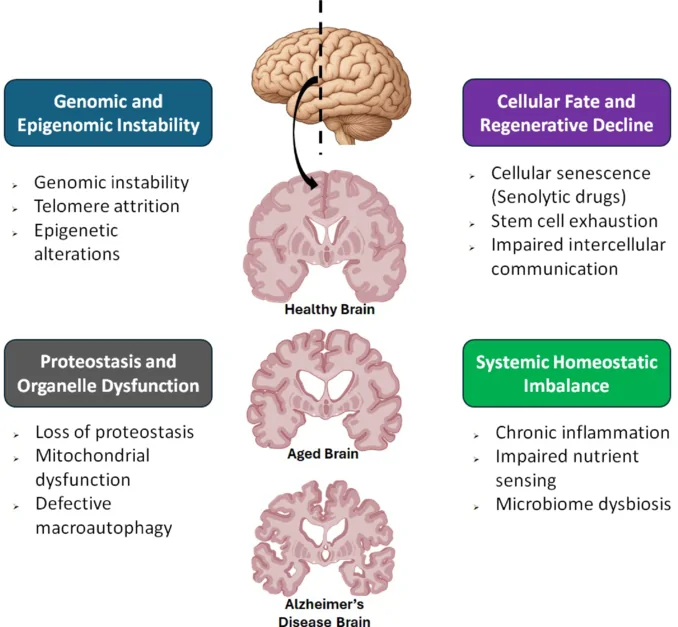
Hallmarks of aging as a functional framework for understanding Alzheimer’s disease progression. This integrative diagram presents four interconnected biological clusters summarizing the aging-associated mechanisms that drive neurodegeneration and Alzheimer’s disease progression. Cluster I: Genomic & epigenomic instability; cluster II: proteostasis & organelle dysfunction; cluster III: cellular fate & regenerative decline; cluster IV: systemic homeostatic imbalance. These clusters provide a conceptual framework linking fundamental aging mechanisms with Alzheimer’s disease pathophysiology, facilitating targeted rejuvenation-based therapeutic strategies. Created with BioRender.com.

The aging process is orchestrated by a complex interplay between molecular and cellular dysfunctions. Historically, this was categorized into a growing list of distinct “hallmarks” (Lopez-Otin et al., 2023). While each hallmark represents a critical mechanism contributing to organismal aging, their sheer number and conceptual overlap can obscure their functional interrelationships and translational significance. To provide a clearer and more integrative perspective, especially in the context of cognitive decline and neurodegenerative diseases, we organized the commonly accepted hallmarks of aging into four functional clusters (**Figure 1**). Each cluster captures a coherent biological domain of aging that not only shares mechanistic foundations but also points toward potential shared therapeutic targets. This clustering aims to enhance the conceptual clarity for researchers and clinicians, while framing the groundwork for rejuvenation strategies that address aging and cognitive decline as a systemic, interconnected process. While earlier reviews have comprehensively covered the full spectrum of aging hallmarks, this review focuses on recent advancements in the field in the context of aging and AD mechanisms, along with a promising strategy capable of targeting these mechanisms systemically.

### Cluster I: Genomic and epigenomic instability

The aging process initiates at the core of biological information, the genome and its regulatory mechanisms. This cluster encompasses genomic instability, telomere attrition, and epigenetic alteration that collectively undermine the integrity, fidelity, and regulation of gene expression over time. Accumulation of DNA damage, progressive shortening of telomeres, and widespread changes in chromatin architecture lead to transcriptional noise, loss of cellular identity, and increased vulnerability to transformation and senescence. These changes are particularly harmful in long-lived, non-replicative tissues such as the brain. In the brain, even subtle shifts in gene regulation can impair neuronal function and its plasticity. Understanding how these core mechanisms decline with age is critical to deciphering the molecular basis of cognitive decline and designing interventions that can restore homeostasis in gene regulation.

#### Genomic instability: Accumulation of somatic mutations and DNA damage due to oxidative stress and impaired repair pathways

Neurons are typically post-mitotic, long-lived cells, with high metabolic activity, which makes them particularly susceptible to the buildup of DNA repair deficiencies over time. Analyses of post-mortem human brain tissue have revealed that neurons, along with glial cells, begin to accumulate DNA double-strand breaks early during AD progression (Thadathil et al., 2021; Provasek et al., 2022). A recent study used single-nucleus RNA sequencing to analyze human and mouse brains and found that excitatory neurons in AD exhibit increased somatic gene fusions, especially those with DNA damage and senescence signatures (Dileep et al., 2023). In this mouse model, neurons with DNA double-strand breaks also showed structural genome changes, elevated cohesin levels, and disrupted 3D genome organization, linking genome instability to neurodegeneration (Dileep et al., 2023). Zhou et al. (2025) showed that single-cell whole-genome sequencing of neurons from AD brains demonstrated a significantly increased burden of somatic single nucleotide variants and short insertion/deletion mutations compared to neurotypical controls. A specific mutational signature associated with oxidative damage and topoisomerase 1-linked mutagenesis was present in 65% of AD neurons but only 5% of controls. These disease-associated short insertion/deletion mutations, primarily two-base pair deletions, suggest that topoisomerase 1-related genome instability is a prominent feature in AD and may contribute to the shared mechanisms of neurodegeneration across multiple disorders (Zhou et al., 2025). Collectively, these findings highlight genomic instability as a pivotal and early feature of AD pathogenesis. The accumulation of diverse forms of DNA damage (e.g., oxidative lesions, double-strand breaks, somatic single nucleotide variants, and short insertion/deletion mutations), as well as genome rearrangements, reflects the compromised DNA repair capacity of aging neurons. Mechanisms such as topoisomerase dysfunction, aberrant activity of DNA repair enzymes, and persistent replication stress contribute to this mutational burden.

#### Telomere attrition: Progressive shortening of telomeres leading to cellular senescence and reduced replicative capacity

Telomeres, the protective DNA protein structures at the ends of chromosomes, progressively shorten with each round of cell division, due to impairments in the function of DNA polymerases to fully replicate the very ends of linear DNA strands, a limitation known as the end-replication problem. This shortening is a hallmark of cellular aging and contributes to replicative senescence, genomic instability, and impaired tissue regeneration (Lopez-Otin et al., 2013; Blackburn et al., 2015). Critically short telomeres can trigger DNA damage responses and limit the proliferative capacity of stem and progenitor cells, thereby compromising tissue homeostasis and repair mechanisms during aging (Jaskelioff et al., 2011). Telomere attrition has been implicated in various age-related diseases, including cardiovascular disease, immune dysfunction, and neurodegeneration (Armanios and Blackburn, 2012), highlighting its central role in the biology of aging.

Multiple recent analyses suggest a complex and nuanced relationship between telomere shortening and AD. Cross-sectional analyses have consistently shown that AD patients exhibit accelerated telomere attrition compared to controls, with shorter telomere length correlating with age, disease duration, and, in some cases, female sex or absence of *APOE4* alleles (Liu et al., 2016). However, results on its association with cognitive performance remain inconsistent, as some data indicate a paradoxical association between shorter telomeres and better cognitive scores in AD patients (Liu et al., 2016). Longitudinal data from the ADNI cohort (Clinical trials identifier: NCT00106899) did not find significant differences in baseline telomere length or its change across diagnostic groups, although converters to mild cognitive impairment/AD showed a non-significant trend toward greater shortening (Nudelman et al., 2019). Additional studies focusing on critically short telomeres found associations with white matter microstructure and plasma neurofilament light levels in healthy older adults, particularly in APOE4 carriers, suggesting telomere erosion may increase vulnerability to neurodegeneration rather than directly mark AD pathology (Lehodey et al., 2024). Finally, Mendelian randomization analysis supports a potential causal role for shorter telomeres in increasing AD risk (Guo and Yu, 2019). Together, these findings indicate that telomere shortening may contribute to susceptibility to AD and progression, although its utility as a reliable biomarker remains uncertain and likely to be context dependent.

#### Epigenetic alterations in Alzheimer’s disease

Epigenetic dysregulation is a key feature of AD, linking genetic susceptibility, environmental exposure, and aging to neuronal dysfunction and neuroinflammation. Recent analyses highlight alterations in histone acetylation, DNA hydroxy-methylation, chromatin accessibility, and retrotransposon activation across multiple brain and immune cell types. Nativio et al. (2020) identified increased H3K27ac and H3K9ac marks in AD postmortem brains, correlating with upregulated chromatin and transcriptional regulators; functionally, these marks exacerbated neurodegeneration in a Drosophila model. Zhou et al. (2025) found that immature granule cells persist into adulthood but decline in AD, accompanied by altered transcriptional profiles, suggesting disrupted neurogenesis linked to epigenetic aging. A recent study has demonstrated that peripheral immune cells are also affected. Ramakrishnan et al. (2024) used single-cell RNA sequencing to reveal AD-specific chromatin signatures in CD8^+^ T cells and monocytes, including RELA-bound elements near *NFKB2*. APOE genotype further influenced these patterns. Similarly, Armstrong et al. (2023) showed that loss of the DNA demethylase TET1 in 5×FAD mice increased amyloid burden and disrupted expression of neuronal and myelination genes, with *TET1* variants enriched in early-onset AD.

The other two recent studies implicate retrotransposon reactivation in AD pathogenesis. LINE-1 ORF1p is an RNA-binding protein encoded by LINE-1 retrotransposons that is normally silenced in somatic tissues but becomes reactivated under pathological conditions. Roy et al. (2024) reported increased LINE-1 ORF1p in microglia from AD brains, which correlated with disease-associated morphology and impaired Aβ phagocytosis; CRISPR activation of LINE-1 in induced pluripotent stem cells (iPSC)-derived microglia induced inflammatory gene programs and AD risk gene expression (Roy et al., 2024). In parallel, Ochoa et al. (2023) demonstrated that pathogenic tau induces heterochromatin decondensation and activates transposable elements, generating double-stranded RNA that drives neuroinflammation in human samples, mice, and Drosophila. Collectively, these findings position epigenetic dysfunction, including histone modifications, methylation defects, and transposon activation, as central to AD pathology across brain and immune compartments, offering promising therapeutic targets.

### Cluster II: Proteostasis and organelle dysfunction

AD is characterized by progressive cognitive decline and neuropathological hallmarks that include extracellular Aβ plaques and intracellular neurofibrillary tangles composed of hyperphosphorylated tau protein. Increasing evidence suggests that impaired proteostasis and organelle dysfunction are central contributors to AD pathogenesis.

#### Loss of proteostasis in Alzheimer’s disease

The failure of protein quality control systems, comprising the ubiquitin-proteasome system (UPS), autophagy-lysosomal pathway, and molecular chaperones, leads to the accumulation of misfolded and aggregation-prone proteins such as Aβ and tau (Ciechanover and Kwon, 2015; Hipp et al., 2019). Disruption of the UPS plays a central role in the early and progressive accumulation of misfolded proteins in AD. In dominantly inherited AD, cerebrospinal fluid levels of UPS components, including E2/E3 ligases and ubiquitin-modifying enzymes, rise up to two decades before symptom onset, correlating with amyloid and tau biomarkers, and inversely with neurodegeneration imaging metrics, suggesting early UPS activation in response to proteotoxic stress and a potential role in tau tangle formation and disease progression (Liu et al., 2025). Mechanistic studies show that a mutant ubiquitin variant, UBB+1, promotes Aβ and tau pathology by interfering with deubiquitination, leading to APP accumulation and increased Aβ secretion, while its silencing reverses these effects in 3D human neural cultures (Maniv et al., 2023). Additionally, TRIM11, a UPS-associated protein with chaperone and disaggregase activity, is downregulated in AD; restoring its expression reduces tau aggregation, neuroinflammation, and cognitive impairment in preclinical models (Zhang et al., 2023b). A sex-specific vulnerability has also been uncovered, where elevated levels of USP11 in females enhance tau pathology via deubiquitination-dependent acetylation, highlighting a novel link between sex and proteostasis-mediated neurodegeneration (Yan et al., 2022). Together, these findings reinforce UPS as a crucial regulator of AD pathology and a promising therapeutic target.

#### Mitochondrial dysfunction in Alzheimer’s disease

Mitochondrial dysfunction, including reduced oxidative phosphorylation, altered dynamics, and excessive reactive oxygen species production, is evident early in AD and contributes to synaptic failure and cell death (Swerdlow, 2018). Additionally, endoplasmic reticulum stress and impaired endoplasmic reticulum-mitochondria signaling interfere with calcium homeostasis and proteostasis, amplifying neurotoxicity (Scheper and Hoozemans, 2015; Area-Gomez and Schon, 2017).

Recent advances highlight mitochondrial dysfunction as a central and multifaceted driver of AD pathogenesis. Astrocytic mitochondrial failure disrupts fatty acid oxidation, leading to lipid droplet accumulation, STAT3-driven astrocyte reactivity, microglial activation, and impaired myelin biosynthesis, ultimately triggering neurodegeneration that mirrors AD phenotypes (Mi et al., 2023). Revisiting the mitochondrial cascade hypothesis, mitochondrial dysfunction is proposed as a primary cause of AD, upstream of amyloid pathology and neuronal failure (Ashleigh et al., 2023). Therapeutically, boosting NAD^+^ with nicotinamide mononucleotide enhances the mitochondrial unfolded protein response via ATF4, restoring synaptic integrity and mitigating neuronal loss in AD models (Xiong et al., 2024). Bioinformatics analyses further identify mitochondria-related hub genes, such as *OPA1* and *TRAP1*, linking mitochondrial metabolism to immune cell infiltration and neuronal vulnerability, with OPA1 shown to protect against Aβ-induced apoptosis (Zhang et al., 2023a). Additionally, NOX4-mediated oxidative stress induces astrocyte ferroptosis by impairing mitochondrial respiration and elevating lipid peroxidation, contributing to neurotoxicity in AD (Park et al., 2021). Together, these findings underscore mitochondrial dysfunction not merely as a downstream consequence but as a potentially pivotal upstream contributor to AD pathogenesis, highlighting it as a promising, though still actively investigated, target for early therapeutic intervention.

#### Impaired autophagy and lysosomal clearance

Impaired autophagy and lysosomal clearance are central features of AD pathology, dysregulated autophagy contributes to defective clearance of protein aggregates and damaged organelles, exacerbating neuronal stress and degeneration (Nixon, 2013). In healthy neurons, autophagy mediates the degradation of damaged proteins and organelles via fusion of autophagosomes with lysosomes. In AD, this process is disrupted at multiple levels: autophagosome clearance is inefficient due to defective lysosomal function, and impaired trafficking and acidification prevent the degradation of Aβ, tau, and damaged mitochondria. These defects lead to the accumulation of autophagic vesicles, particularly in dystrophic neurites surrounding amyloid plaques. Genetic mutations in *PSEN1* have been shown to impair lysosomal acidification and autophagic flux, further linking defective proteostasis with familial AD. Therapeutic activation of autophagy-related pathways, such as transcription factor EB (TFEB)-mediated lysosomal biogenesis, is being explored to restore clearance mechanisms and mitigate neurodegeneration in AD (Nilsson et al., 2013; Lee, 2022).

Recent analyses emphasize the pivotal role of autophagy in mitigating AD pathology. Hederagenin, a plant-derived triterpene, was shown to ameliorate AD symptoms by promoting autophagic flux through the peroxisome proliferator-activated receptor alpha (PPARα)/TFEB signaling axis, leading to reduced Aβ deposition and cognitive improvement in APP/PS1 mice and *C. elegans* models (Xie et al., 2023). Similarly, inhibition of miR-331-3p and miR-9-5p restored autophagy and enhanced Aβ clearance in late-stage AD mice by derepressing autophagy-related targets, suggesting potential for stage-specific therapeutic intervention (Chen et al., 2021). Astrocyte-specific autophagy was found to be dynamically responsive to Aβ, and its modulation, particularly via LC3B and SQSTM1, directly influenced plaque burden and cognitive performance in APP/PS1 mice (Kim et al., 2024). Furthermore, magnolol, a bioactive compound, improved AD-like pathologies by activating autophagy through the AMP-activated protein kinase/mammalian target of rapamycin /ULK1 pathway, thereby inhibiting apoptosis and enhancing cognitive outcomes (Wang and Jia, 2023). Lastly, microglial autophagy was shown to be essential for amyloid engagement and prevention of microglial senescence, reinforcing its role in sustaining immune surveillance in the AD brain (Choi et al., 2023; Tang et al., 2024). Collectively, these findings establish autophagy enhancement as a converging mechanism across diverse cellular targets (e.g., neurons, astrocytes, and microglia in combatting AD).

### Cluster III: Cellular fate and regenerative decline in Alzheimer’s disease

AD is marked not only by progressive neurodegeneration but also by a profound impairment in the brain’s endogenous regenerative capacity. In healthy adult brains, neurogenesis persists in limited regions such as the hippocampal dentate gyrus and the subventricular zone; however, this process is markedly suppressed in AD due to altered niche signaling, chronic inflammation, and age-related stem cell exhaustion (Mu and Gage, 2011; Winner and Winkler, 2015). Neural stem and progenitor cells in AD exhibit impaired proliferation, disrupted fate specification, and a shift toward gliogenic rather than neurogenic outcomes, further exacerbating cognitive decline (Katsimpardi et al., 2014; Moreno-Jimenez et al., 2019). Additionally, cellular senescence and mitochondrial dysfunction in both neural and glial lineages contribute to a hostile environment that hinders neuronal regeneration and synaptic repair (Martinez-Cue and Rueda, 2020). Moreover, altered intercellular signaling, particularly involving inflammatory cytokines, extracellular vesicles, and impaired trophic support, disrupts the homeostatic crosstalk necessary for neural resilience and repair (Franceschi et al., 2018). Understanding how cellular fate decisions are dysregulated in AD offers a critical window into designing regenerative therapies aimed at restoring neuroplasticity and cognitive function.

#### Cellular senescence in Alzheimer’s disease

Cellular senescence, a state of stable cell cycle arrest accompanied by a pro-inflammatory secretory profile, is increasingly implicated in the pathogenesis of AD. Senescent cells accumulate in the aging brain, including astrocytes, microglia, oligodendrocyte progenitors, and even neurons, where they adopt a senescence-associated secretory phenotype (SASP) that promotes chronic neuroinflammation and neuronal dysfunction (Bussian et al., 2018; Musi et al., 2018). The SASP contributes to a toxic milieu by releasing cytokines (e.g., interleukin [IL]-6, IL-1β), proteases, and reactive oxygen species, which amplify tau pathology, impair synaptic plasticity, and disrupt neural networks (Riessland et al., 2019; Walton and Andersen, 2019). Clearance of senescent glial cells in mouse models of tauopathy improves cognitive function and reduces pathology, underscoring their causal role and therapeutic relevance (Bussian et al., 2018). These findings position cellular senescence as a key mechanism driving regenerative decline and neurodegeneration in AD.

Recent analyses provide compelling evidence that cellular senescence contributes to AD pathology, particularly through glial cell dysfunction. Single-nucleus RNA sequencing and imaging mass cytometry of postmortem AD brains revealed widespread premature senescence in microglia, marked by impaired phagocytosis, DNA damage, and elevated senescence-associated markers (Fancy et al., 2024). Pilot clinical trials using the senolytics dasatinib and quercetin demonstrated central nervous system penetration, safety, and preliminary signs of target engagement, supporting further exploration of senolytic therapies in early AD (Gonzales et al., 2022). Additionally, microglial senescence in aging and AD was linked to elevated lactate-induced H3K18 lactylation, which promoted nuclear factor κB activation and SASP expression, identifying a novel epigenetic driver of neuroinflammation (Wei et al., 2023). Mechanistically, astrocyte senescence was shown to be driven by YAP-CDK6 pathway inhibition, with pharmacological YAP activation reversing senescent phenotypes and improving cognition in AD models (Xu et al., 2021). Together, these findings underscore glial senescence as a central mechanism in AD and support the therapeutic potential of targeting senescent cells.

#### Stem cell exhaustion in Alzheimer’s disease

Stem cell exhaustion, a hallmark of aging, is increasingly recognized as a critical contributor to the pathophysiology of AD. In the aging brain, a progressive decline in neural stem progenitor cell proliferation and neurogenesis has been documented, particularly within the hippocampal dentate gyrus, a region essential for learning and memory (Katsimpardi et al., 2014; Moreno-Jimenez et al., 2019). This decline is exacerbated in AD, where pathological hallmarks such as Aβ deposition, chronic inflammation, and oxidative stress create a hostile microenvironment that impairs neural stem progenitor cell maintenance, differentiation, and survival (Mu and Gage, 2011). Moreover, studies using transgenic AD mouse models have shown accelerated depletion of neural stem progenitor cells, often accompanied by cellular senescence and loss of neurogenic potential (Melo Dos Santos et al., 2024). The failure of endogenous regenerative capacity due to stem cell exhaustion may not only limit repair mechanisms but also contribute to cognitive decline, positioning stem cell preservation and rejuvenation as a promising therapeutic avenue in AD.

Emerging evidence highlights that both intrinsic and extrinsic disruptions to neural stem cell (NSC) function contribute significantly to AD pathology. Familial AD-associated mutations in *PSEN1* have been shown to impair Notch signaling and accelerate premature neurogenesis in iPSC-derived models and postmortem tissue, reflecting a stem cell aging phenotype (Arber et al., 2021). This is compounded by AD-associated neuroinflammation, where disease-associated microglia and reactive astrocytes exacerbate NSC dysfunction. Notably, extracellular vesicles (EVs) derived from human iPSC-NSCs exhibit neuroprotective and anti-inflammatory effects in 5×FAD mice, reversing microglial and astrocytic activation and reducing Aβ and p-tau levels (Madhu et al., 2024). Furthermore, cell-type-specific EV proteomic profiling revealed astrocyte-derived EVs enriched in AD brains, implicating integrin-β1 as a potential driver of AD progression (You et al., 2022). Mechanistically, miR-132, a microRNA consistently downregulated in AD, was identified as a critical regulator of adult hippocampal neurogenesis, and its restoration reversed neurogenic and cognitive deficits in AD models (Walgrave et al., 2021). Together, these studies underscore that therapeutic strategies targeting NSC dysfunction, whether through modulation of signaling pathways, EV-based interventions, or miRNA replacement, offer promising avenues for altering the course of AD.

#### Altered intercellular communication

In AD, intercellular communication is profoundly disrupted, contributing to synaptic dysfunction, neurodegeneration, and chronic neuroinflammation (De Strooper and Karran, 2016). One major axis of disturbance involves glial–neuronal crosstalk: reactive astrocytes and activated microglia release excessive cytokines (e.g., IL-1β, tumor necrosis factor α) and complement proteins (e.g., C1q), which impair synaptic function and promote neuronal injury. Exosomal signaling is also altered, with AD brains showing changes in the cargo of extracellular vesicles, including increased tau and Aβ species, which may propagate pathology (Rajendran et al., 2014). Moreover, senescent cells in the aging brain adopt a SASP, releasing inflammatory mediators and matrix-remodeling enzymes that disrupt the extracellular environment and impair neurovascular unit integrity (Bussian et al., 2018). Dysregulation of neurotransmitter systems, including glutamate and acetylcholine, further reduces effective neuronal communication. In AD, excessive glutamatergic signaling contributes to excitotoxicity and synaptic loss, while cholinergic deficits, particularly in the basal forebrain, are strongly associated with cognitive decline and memory impairment (Francis et al., 1999; Wang and Reddy, 2017). Together, these alterations compromise the homeostatic balance and contribute to the progressive decline in cognitive function characteristic of AD.

Recent findings have revealed that altered intercellular communication is a central and multifaceted contributor to AD pathology. Brain-resident CD8⁺ T cells, orchestrated by CXCR6-CXCL16 signaling with microglia, exhibit a protective role by limiting Aβ deposition and neuroinflammation, highlighting a neuroimmune axis critical for maintaining tissue homeostasis (Su et al., 2023). Conversely, tunneling nanotubes have emerged as conduits for the bidirectional transfer of toxic α-synuclein aggregates and mitochondria between neurons and microglia, suggesting both pathogenic and compensatory intercellular trafficking in neurodegeneration (Chakraborty et al., 2023). Furthermore, microglial activation via the cGAS-STING pathway exacerbates amyloid pathology and disrupts intercellular signaling, while its targeted deletion preserves neuroprotective communication and mitigates disease progression (He et al., 2025). Single-nucleus transcriptomics in human AD brains have identified widespread dysregulation of ligand-receptor interactions, particularly impairing astrocyte-to-neuron signaling through pathways involving APOE and CALM (Liu et al., 2024). Spatial transcriptomics further underscores the impact of microglia-astrocyte crosstalk within amyloid plaque niches, revealing that heightened microglial density induces neurotoxic astrocytic phenotypes and disrupts excitatory-inhibitory balance in neurons (Mallach et al., 2024). Collectively, these findings highlight the importance of preserving or restoring intercellular communication networks as a therapeutic strategy in AD.

### Cluster IV: Systemic homeostatic imbalance in Alzheimer’s disease

While AD is primarily characterized by neuropathological hallmarks such as amyloid-beta plaques and tau tangles, it is increasingly recognized as a systemic disorder marked by widespread homeostatic imbalance. Aging disrupts the equilibrium of multiple physiological systems; immune, endocrine, metabolic, vascular, and microbial that collectively sustain brain health. Chronic low-grade inflammation, termed “inflamm-aging,” is a key systemic alteration that exacerbates neurodegeneration, with AD patients displaying elevated peripheral pro-inflammatory cytokines that may contribute to central neuroinflammation via impaired blood–brain barrier (BBB) function (Franceschi et al., 2018). Similarly, systemic insulin resistance and altered insulin-like growth factor 1 signaling are linked to impaired neuronal glucose metabolism, increased oxidative stress, and amyloidogenic APP processing (de la Monte, 2017). The gut–brain axis is also implicated, as age-associated dysbiosis influences peripheral immunity and produces microbial metabolites, such as lipopolysaccharides, that reach the brain and amplify amyloid and inflammatory cascades (Zhao et al., 2017). Additionally, vascular aging contributes to cerebral hypoperfusion and reduced clearance of amyloid and metabolic waste, reinforcing neurodegenerative processes (Sweeney et al., 2019). Together, these systemic dysfunctions suggest that restoring organism-wide homeostasis may be essential for mitigating or preventing AD progression.

#### Chronic inflammation in Alzheimer’s disease

Chronic inflammation is a central contributor to the pathogenesis and progression of AD. In both aging and AD, there is an upregulation of innate immune activity, particularly microglial and astrocyte-mediated responses to accumulating Aβ and tau pathology (Heneka et al., 2015). While initially protective, prolonged activation of glial cells results in sustained release of pro-inflammatory cytokines such as IL-1β, tumor necrosis factor α, and IL-6, which exacerbate neuronal injury and synaptic dysfunction (Heppner et al., 2015). Furthermore, genetic studies highlight a strong link between immune pathways and AD risk, including variants in TREM2 and CD33, underscoring the pathogenic role of dysregulated immune responses (Griciuc et al., 2013). Peripheral inflammation also feeds into central pathology, suggesting that AD involves both local and systemic inflammatory components that contribute to a vicious cycle of neurodegeneration.

Sangineto et al. (2023) demonstrated that microglia in AD adopt a hypermetabolic state with dysfunctional mitochondria and elevated reactive oxygen species, which can be reversed by inhibiting succinate dehydrogenase using dimethyl malonate, thereby suppressing inflammation via HIF-1α and glycolysis inhibition in 3×Tg-AD mice. Complementing this metabolic-inflammation link, Chen et al. (2021) showed that folic acid and vitamin B12 supplementation improved cognition and decreased homocysteine and tumor necrosis factor α levels in AD patients, suggesting that modulating the one-carbon metabolism may attenuate neuroinflammation. Liu et al. (2022) identified transcription factors, including ZNF384, that modulate shared inflammatory-metabolic pathways in psoriasis and AD, proposing ZNF384 as a novel target bridging systemic inflammation and neurodegeneration. Another study employed translocator protein-positron emission tomography imaging and showed that brain inflammation in early-onset AD co-localizes more strongly with tau than with amyloid or atrophy, reinforcing the role of inflammation in tau propagation and cognitive impairment (Appleton et al., 2025). Supporting this, Dutta et al. (2023) revealed that tau fibrils activate microglia through the TLR2/MyD88/nuclear factor κB pathway, and inhibiting this axis with a peptide reduced gliosis, tau pathology, and cognitive decline in PS19 mice, a tauopathy mouse model. Finally, Lopez-Rodriguez et al. (2021) highlighted the vulnerability of AD brains to systemic inflammatory insults; IL-1β induced amplified astrocytic chemokine responses and neuronal network dysfunction in APP/PS1 mice and human AD tissue, underscoring the detrimental synergy between systemic inflammation and AD-related priming. Together, these studies converge on a critical theme: inflammation, particularly when driven by specific metabolic or innate immune pathways, amplifies tau pathology and cognitive dysfunction, offering viable avenues for therapeutic intervention.

#### Impaired nutrient sensing in Alzheimer’s disease

AD is increasingly recognized as a disorder involving disrupted nutrient sensing pathways, particularly those regulating cellular metabolism and aging. Central among these are the insulin/insulin-like growth factor 1 signaling pathway, AMP-activated protein kinase, and the mammalian target of rapamycin, which coordinate cellular responses to nutrient availability and energy stress. In AD brains, insulin resistance is well-documented, impairing neuronal glucose uptake and contributing to synaptic dysfunction and tau hyperphosphorylation (Talbot et al., 2012). Dysregulation of mammalian target of rapamycin signaling, particularly its hyperactivation, has been implicated in impaired autophagy and increased Aβ and tau pathology (Tramutola et al., 2015). Concurrently, reduced AMP-activated protein kinase activity undermines cellular energy homeostasis and antioxidant responses, further exacerbating neurodegeneration (Vingtdeux et al., 2011). These disruptions in nutrient-sensing pathways not only accelerate the aging phenotype in the brain but also hinder the activation of protective stress responses, positioning them as critical nodes for therapeutic intervention in AD.

Recently, nutrient-sensing neurons and neuroactive compounds that target metabolic architecture, particularly those influencing glycolysis and ketone/lipid metabolism, appear capable of delaying age-related diseases and AD-like pathology, underscoring the therapeutic potential of Creb-binding protein pathways in specific neurons (Litke et al., 2022). Complementing this, γ-secretase activity in neurons was shown to regulate cholesterol metabolism, with its chronic suppression impairing synaptic function via reduced neuronal cholesterol, highlighting a mechanistic link between lipid metabolism and synaptic deficits in AD (Essayan-Perez and Sudhof, 2023). Clinically, blood-based biomarkers such as neurofilament light chain and glial fibrillary acidic protein, but not amyloid or tau species, were associated with cognitive decline and incident dementia in individuals with diabetes and obesity, suggesting utility for tracking neurodegeneration in metabolically at-risk populations (Mielke et al., 2025). Large-scale metabolomics studies identified numerous plasma metabolites, including cholesterol subfractions in low- and high-density lipoproteins, as predictive of AD and vascular dementia, with high predictive accuracy when integrated with cognitive and demographic data (Huang et al., 2023; Qiang et al., 2024). Finally, GLP-1 receptor agonists and SGLT2 inhibitors, two classes of antihyperglycemic drugs, demonstrated significant prophylactic benefits against neurodegenerative diseases, suggesting metabolic interventions may have broad neuroprotective effects (Tseng et al., 2025). Together, these findings converge on a metabolic-inflammation-synapse axis as a key vulnerability in AD, offering diverse therapeutic and diagnostic avenues.

#### Microbiome dysbiosis in Alzheimer’s disease

Microbiome dysbiosis has emerged as a key contributor to AD pathophysiology through its modulation of systemic inflammation, immune responses, and brain homeostasis. Alterations in gut microbial composition, characterized by reduced microbial diversity and an overrepresentation of pro-inflammatory taxa such as *Escherichia/Shigella*, have been linked to elevated peripheral inflammation and BBB permeability, facilitating neuroinflammatory cascades that accelerate Aβ deposition and tau pathology (Cattaneo et al., 2017; Vogt et al., 2017). Furthermore, microbial metabolites, such as short-chain fatty acids, trimethylamine-N-oxide, and lipopolysaccharide, influence neuroimmune signaling, microglial activation, and mitochondrial function, contributing to neurodegeneration (Tran and Mohajeri, 2021; Loh et al., 2024). Experimental models confirm that gut microbiota depletion or modulation can alter AD-related pathology and cognitive performance, supporting the gut-brain axis as a potential therapeutic target (Dodiya et al., 2019).

**Figure 2:**
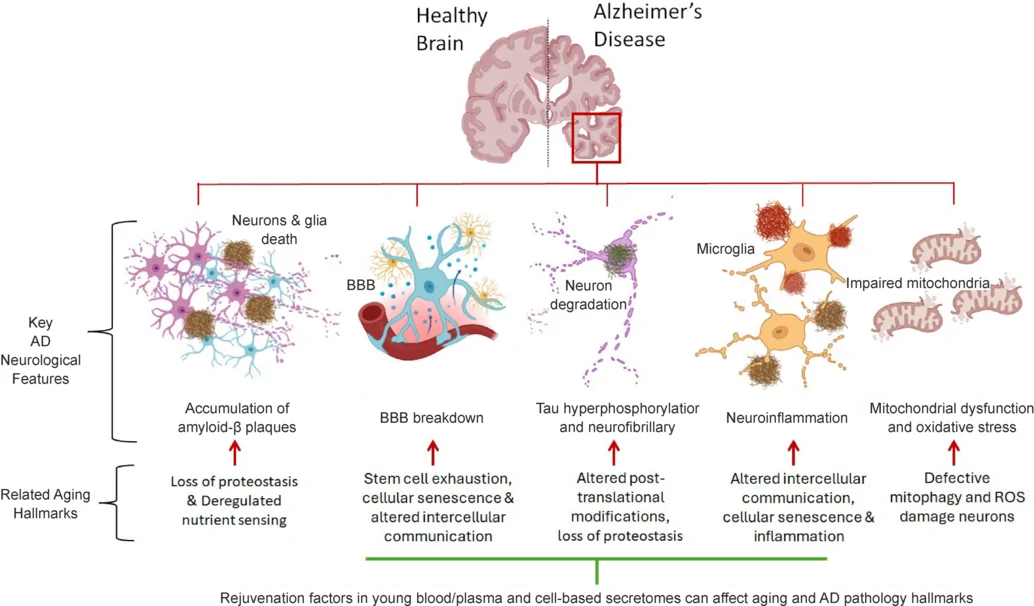
Neurodegenerative signatures of AD aligned with hallmarks of aging. The upper panel shows a comparative illustration of healthy brain tissue *versus* AD-affected brain tissue, with the red box highlighting the region of pathological changes. The lower panel depicts five major neuropathological features characteristic of AD; amyloid-β plaque accumulation, BBB breakdown, tau hyperphosphorylation and neurofibrillary tangles, neuroinflammation with activated microglia, and mitochondrial dysfunction. Each feature is linked to specific aging hallmarks including proteostasis loss, cellular senescence, altered intercellular communication, and oxidative stress. The green bar at the bottom indicates that rejuvenation factors present in young blood/plasma and cell-based secretomes that may influence both aging processes and AD pathology hallmarks. Created with BioRender.com. AD: Alzheimer’s disease; BBB: blood–brain barrier; ROS: reactive oxygen species.

Emerging evidence supports a critical role of gut microbiome dysbiosis in the onset and progression of AD. In a cross-sectional study of cognitively normal individuals, those with biomarker evidence of preclinical AD displayed a distinct gut microbial profile correlated with Aβ and tau pathology, suggesting gut microbiome alterations preceding cognitive symptoms (Ferreiro et al., 2023). A meta-analysis further confirmed gut dysbiosis in AD and mild cognitive impairment, revealing decreased microbial diversity and region-specific alterations in taxa such as *Bacteroides* and *Phascolarctobacterium* (Jemimah et al., 2023). Mechanistic insights from animal models show that recolonization of germ-free AD mice with microbiota from AD patients exacerbates neuropathological features and neuroinflammation through pathways involving polyunsaturated fatty acids and the C/EBPβ-asparagine endopeptidase axis (Chen et al., 2022). Specific microbial strains, such as *Faecalibacterium prausnitzii*, which are reduced in mild cognitive impairment and AD patients, have been shown to improve cognitive function and reduce oxidative stress in mouse models when reintroduced (Ueda et al., 2021). Notably, fecal microbiota transplants from AD patients impaired hippocampal neurogenesis and cognitive behaviors in healthy rats, highlighting a causal role for gut microbiota in AD-related deficits (Grabrucker et al., 2023). Finally, a human-origin probiotics cocktail significantly attenuated AD pathology and cognitive decline in APP/PS1 mice by reducing gut and brain inflammation, preserving tight junctions, and restoring microbial balance (Prajapati et al., 2025). Collectively, these findings underscore the influence of the gut microbiome on AD pathophysiology and its potential as a diagnostic and therapeutic target. **Figure 2** summarizes the neurodegenerative signatures of AD that are aligned with the functional clustering of aging.

## Injectable Rejuvenating Biologics

While symptomatic treatments such as cholinesterase inhibitors (e.g., donepezil, rivastigmine) and NMDA receptor antagonists (e.g., memantine) have been widely used to manage cognitive and behavioral symptoms, recent therapeutic developments have shifted toward disease-modifying strategies, with current U.S. Food and Drug Administration-approved therapies largely centered on amyloid-targeting monoclonal antibodies, such as lecanemab and donanemab (Mintun et al., 2021; van Dyck et al., 2023). While these agents reduce amyloid burden and may modestly slow cognitive decline, they do not directly address the broader hallmarks of aging, neuroinflammation, or neuronal loss. A growing body of evidence supports the concept that targeting the fundamental hallmarks of aging might offer broader and more effective therapeutic outcomes in AD (Lopez-Otin et al., 2013; Hou et al., 2019). Interventions designed to modulate these hallmarks include several approaches such as senolytic drugs (Zhang et al., 2019), autophagy enhancers (Xie et al., 2023), epigenetic reprogramming agents (Lu et al., 2020), metabolic and signaling modulators such as NAD^+^ precursors (Hou et al., 2018), resveratrol, and small molecules (e.g., NE3107; Reading et al., 2021; Tosatti et al., 2022), all showing preclinical promise in mitigating neurodegenerative changes.

The heterochronic parabiosis studies provided a new approach for targeting multiple mechanisms of action of aging. In this model, the circulatory systems of young and old animals are connected surgically and demonstrate rejuvenation potential in multiple tissues and organs in elder mice (Lopez-Otin et al., 2013). Exposing older mice to young blood reversed cognitive and cellular impairments in aged and AD mouse models (Villeda et al., 2014; Castellano et al., 2017). These effects are probably attributed to the circulating factors present in young blood. These findings have catalyzed scientific interest in developing injectable biologics, which are enriched with rejuvenating factors such as blood, plasma, stem cell–derived secretomes and other derivatives (e.g. exosomes) capable of modifying AD course (**Figure 2**). Some of these regenerative approaches, including plasma transfusions, senolytics, metabolic modulators, cell-based therapies are currently being tested in human clinical trials (**Table 1**). Furthermore, combination paradigms are increasingly considered: for instance, coupling senolytic therapies with secretome-based interventions may both alleviate detrimental cellular senescence and enhance tissue rejuvenation, ultimately improving therapeutic efficacy (Bussian et al., 2018; Gonzales et al., 2023). It is important to note that despite promising preclinical and early clinical evidence, injectable biologics face significant translational challenges. For instance, administration routes vary considerably (**Table 1**), with most plasma-based interventions requiring resource-intensive intravenous infusion and repeated hospital visits, while alternative approaches such as oral or intranasal delivery may increase the compliance of patients. High costs associated with cell therapies and plasma treatments may limit accessibility, highlighting the need for scalable and robust manufacturing, whereas oral small-molecule modulators offer logistical and economic advantages (Lipsitz et al., 2017). Regulatory pathways present additional complexity, as biologics must meet stringent safety and potency requirements through multi-phase trials (Marks et al., 2017). Successful clinical translation, therefore, requires optimization of delivery methods, cost-effectiveness, and regulatory compliance to ensure broader patient access. Among the various injectable biologics under investigation, young blood and plasma are extensively studied as rejuvenating interventions, with their therapeutic potential rooted in the foundation of modifying the hallmark of aging.

**Table 1:** Clinically tested rejuvenation approaches in AD

Rejuvenation approach	Treatment	ROA	MOA	Trial phase	Main results/status	NCT/reference
Senolytic therapy	Dasatinib + Quercetin	Oral	Clears senescent cells and decrease, inflammation	Phase 1 (completed)	First-in-human AD trial showed target engagement/safety signals; exploratory biomarker changes reported; larger efficacy trials needed	NCT04063124/Gonzales et al., 2023
Plasma-based therapy	Young plasma (open-label Stanford PLASMA study)	IV	Supplies youthful circulating factors that may enhance synaptic function & reduce neuroinflammation	Early pilot	Feasible and safe in mild-to-moderate AD; cognitive outcomes exploratory/inconclusive; informed later plasma-fraction programs	NCT02256306/Sha et al., 2019
	Plasma fraction GRF6019 (Alkahest)	IV	Enriched youthful plasma proteins; pro-homeostatic, anti-inflammatory, pro-synaptic effects	Phase 2a (completed)	Double-blind RCT in mild-to-moderate AD: acceptable safety; mixed clinical signals; further optimization required	NCT03520998/Hannestad et al., 2020
	Therapeutic plasma exchange + albumin (AMBAR)	Apheresis + IV	Removes pathogenic plasma factors; albumin replacement may bind Aβ/oxidants and improve transport	Phase 2b/3 (completed)	Post hoc and predefined analyses suggested slowed decline, especially in moderate AD; prompted ongoing discussions on confirmatory studies	NCT01561053/Boada et al., 2020
	Exercise-conditioned plasma (ExPlas)	IV	Donor plasma collected after endurance exercise; delivers exercise-induced rejuvenating factors	RCT (protocol published)	Protocol details published; outcomes pending; positions “exercise plasma” as a mechanistic test of youthful factors	NCT05068830/Tari et al., 2022
Cell–based therpies	Allogeneic MSCs (Lomecel-B / laromestrocel)	IV	Paracrine/secretome-mediated immunomodulation; pro-trophic, pro-vascular effects	Phase 1 (completed); Phase 2 ongoing	Phase-1 AD study reported acceptable safety and feasibility; subsequent studies progressing to define efficacy	NCT02600130/Brody et al., 2023
Metabolic and signaling modulators	Nicotinamide Riboside (NR, NAD^+^ booster)	Oral	Enhances NAD^+^ metabolism, improves mitochondrial function, and reduces neuroinflammation	Phase I (Completed)	Increased NAD^+^ levels in CSF and blood; cognitive readouts exploratory	NCT03482167/Radenkovic et al., 2020
	Resveratrol (SIRT1 activator)	Oral	Activates SIRT1, improves mitochondrial function and reduces amyloid/tau pathology	Phase II	High-dose resveratrol safe; slowed decline in CSF Aβ40, altered MMP9; cognition unchanged	NCT01504854/Turner et al., 2015
	NE3107 (Insulin-sensitizing anti-inflammatory small molecule)	Oral	Reduces neuroinflammation via ERK/NF-κB inhibition and improves insulin sensitivity	Phase III (AD, ongoing)	Promising in Phase II for Parkinson’s; AD results pending	NCT04669028/Haroon et al., 2024

Aβ: Amyloid-beta; AD: Alzheimer’s disease; AMBAR: Alzheimer’s Management by Albumin Replacement (clinical trial name); CSF: cerebrospinal fluid; ERK: extracellular signal-regulated kinase; ExPlas: exercise-conditioned plasma; GRF6019: proprietary plasma fraction product developed by Alkahest; IV: intravenous; Lomecel-B/laromestrocel: allogeneic mesenchymal stem cell (MSC) product names (used by Longeveron); MMP9: matrix metalloproteinase-9; MOA: mechanism of action; MSC: mesenchymal stem cell; NAD^+^: nicotinamide adenine dinucleotide (oxidized form); NF-κB: nuclear factor kappa-light-chain-enhancer of activated B cells; NCT: National Clinical Trial (identifier from ClinicalTrials.gov); NE3107: a small-molecule anti-inflammatory/insulin-sensitizing compound (code name for benzyl ethyl indole-derived drug); NR: nicotinamide riboside; RCT: randomized controlled trial; ROA: route of administration; SIRT1: sirtuin-1 (NAD^+^-dependent deacetylase).

### Rejuvenation potential of young blood and plasma

Recent studies in both animal models and early human clinical trials have provided compelling evidence that young blood or plasma may exert neuroprotective effects relevant to AD. In murine models, intravenous delivery of young blood serum has been shown to attenuate cognitive decline and reduce AD pathology. Xia et al. (2019) demonstrated that young blood restored hippocampal dependent learning and memory in aged APP/PS1 mice, reduced Aβ plaque load, and promoted cholinergic synaptic integrity through activation of neuroprotective REST/FOXO1 signaling. Importantly, pharmacological blockade of hippocampal cholinergic activity diminished these benefits, highlighting the centrality of the cholinergic system in the observed rejuvenation (Xia et al., 2019). Similarly, Middeldorp et al. (2016) found that both parabiosis and plasma injections from young mice restored synaptic protein levels, reversed aberrant extracellular signal-regulated kinase signaling, and improved memory performance in aged AD mice, even without altering amyloid plaque burden. Interesting, Gulej et al. (2024) used heterochronic parabiosis to show that young blood exposure enhanced BBB integrity and increased capillary density in aged mice, while old blood accelerated cerebrovascular aging in young mice. These findings underscore the vascular dimension of AD and suggest that young plasma may help preserve neurovascular function (Gulej et al., 2024). In parallel, mechanistic studies revealed that Aβ amyloids induced pericyte ferroptosis via the CD36/PINK1/Parkin pathway contribute to BBB breakdown in AD models, reinforcing the importance of vascular health (Li et al., 2022c). Hernandez et al. (2023) also reported that young plasma administration reduced phosphorylated tau levels and tangle formation in a tauopathy mouse model, although cognitive improvements were not detected. Additionally, young plasma therapy has shown promise in aged ovariectomized rats with AD, significantly improving cognitive function, reducing oxidative stress, and restoring miR-134a, SIRT-1, CREB, and BDNF expressions in the hippocampus, often matching or surpassing the effects of estrogen therapy (Habibi et al., 2024). Similarly, plasma from exercise-trained donors enhanced neuronal viability *in vitro* and promoted hippocampal neurogenesis in AD rat models, potentially due to reduced pro-inflammatory cytokines, highlighting the role of blood-borne factors in neuroprotection (Norevik et al., 2024).

Recent clinical trials have explored the potential of blood derivatives and plasma-based therapies for AD, focusing on their safety, tolerability, and efficacy. One such trial is the PLASMA study (ClinicalTrials.gov ID: NCT02256306), a randomized, double-blind, crossover trial evaluating the safety and tolerability of young fresh frozen plasma in AD patients. Conducted at Stanford University, the trial enrolled 18 patients with mild-to-moderate AD (Sha et al., 2019). The infusions were well-tolerated and feasible, with no serious adverse events attributed to young fresh frozen plasma. However, the study was underpowered to assess efficacy due to its small sample size and short duration. The Alzheimer’s Management By Albumin Replacement study (Boada et al., 2020; ClinicalTrials.gov ID: NCT01561053) represents the largest randomized controlled trial to date on plasma exchange (PE) with albumin replacement in AD (*n* = 347). PE-treated patients showed slower cognitive and functional decline, particularly in those with moderate AD. Significant benefits were noted in the Alzheimer’s Disease Cooperative Study – Activities of Daily Living score and Clinical Dementia Rating Sum of Boxes, with up to 71% less decline compared to placebo. The treatment was associated with a favorable safety profile. Complementing these findings, Gonzalo et al. (2024; PMID: 39476248, PMCID: PMC11651178) analyzed serum and cerebrospinal fluid (CSF) samples from Alzheimer’s Management By Albumin Replacement trial participants. PE with albumin significantly reduced inflammatory mediators (e.g., interferon-γ, intercellular adhesion molecule 1, macrophage inflammatory protein-1α, and Eotaxin), suggesting that anti-inflammatory mechanisms may underlie its cognitive benefits. In placebo-treated patients, increases in macrophage inflammatory protein-1α correlated with clinical worsening, a relationship not seen in the PE group, further implicating immune modulation in therapeutic efficacy. A novel ongoing pilot trial, ExPlas (Exercised plasma), is exploring plasma transfusions from exercise-trained donors in early AD. The study, currently in progress, is designed to assess safety, cognitive outcomes, and biomarkers related to brain function and inflammation. Its rationale stems from the rejuvenating systemic effects of exercise, which may be transferable via plasma factors (Tari et al., 2022). Finally, another human study found that BBB permeability increases with age, particularly in APOE4 carriers, and is associated with amyloid pathology, suggesting that therapeutic approaches preserving BBB function could be especially valuable in early or preclinical AD (Denkinger et al., 2024). In summary, while early clinical trials suggest plasma-based therapies are safe and biologically active, consistent cognitive benefits remain inconclusive. Moderate-stage AD patients may derive the most benefit, and modulation of peripheral and central inflammation could represent a key mechanism. Larger, longer-term trials with targeted patient selection and mechanistic endpoints are needed to clarify therapeutic potential.

### Rejuvenation potential of cell-based secretomes

While young blood plasma transfusions have demonstrated therapeutic potential to modulate systemic and neuroinflammatory pathways implicated in AD and other neurodegenerative diseases, their clinical efficacy needs further validation (Hosseini et al., 2024; Lee et al., 2024). Furthermore, practical challenges such as donor variability, immunogenicity risks, pathogenic concerns, and scalability issues pose significant barriers to its widespread implementation (Lehallier et al., 2019; Ackfeld et al., 2022). These limitations have prompted growing interest in finding alternative injectable solutions that are safer, more scalable, and robust. Cell-based secretomes (a.k.a. condition media) contain a rich cocktail of bioactive factors, which include growth factors, cytokines, neuroprotective factors, and other proteins encapsulated in extracellular vesicles or free (Li et al., 2022b). The cell source from which the injectable secretome or derivatives (e.g., exosomes) is derived dictates the composition of the injectable solution and its physiological effect (da Silva et al., 2023).

Recent preclinical and clinical studies highlight the therapeutic promise of stem cell-derived secretomes from diverse origins in multiple indications. For example, in musculoskeletal models, secretomes from human umbilical-mesenchymal stem cells (MSCs) significantly improved cartilage repair in sheep, particularly when combined with microfracture (Lubis et al., 2023a), and showed comparable efficacy to MSCs in early osteoarthritis (Lubis et al., 2023b). Similarly, secretome from human dental pulp stem cells promoted structural and radiological improvements in rats with knee osteoarthritis (Lubis et al., 2023b; Nowzari et al., 2023). In aged mice, intramuscular delivery of a stem cell–derived secretome enhanced muscle mass, physical function, and metabolism (Fennel et al., 2024), while subcutaneous injection of adipose stromal cell–secretome improved renal recovery and reduced inflammation in rats with acute kidney injury (Ullah et al., 2024). These regenerative effects are now entering the clinical stage. In the first-in-human case of the SECRET-HF trial, repeated intravenous infusion of a cardiovascular progenitor cell-derived, EV-enriched secretome in a heart failure patient was safe, well-tolerated, and associated with improved cardiac function, reduced diuretic use, and no immunogenic response (Menasche et al., 2024). These results support the concept that secretome therapy may act via immune modulation to promote tissue repair across diverse conditions.

The secretome of different types of stem cells derived cells demonstrated that the secretomes derived from NSCs, bone marrow mesenchymal stem cells (BM-MSCs), dental pulp stem cells, and adipose-derived stem cells (ASCs) can modulate key pathological features of neurodegenerative diseases and conditions such as Parkinson’s disease, AD, and spinal cord injury. In Parkinson’s disease models, NSC-derived conditioned medium (NSC-CM) protected dopaminergic neurons from 6-hydroxydopamine induced mitochondrial damage, reduced oxidative stress and apoptosis, and improved motor function *in vivo*. These effects were partly mediated by PARK7 and the activation of SIRT1 signaling (Ni et al., 2022). Similarly, secretome from BM-MSCs promoted dopaminergic neuron survival more effectively than cell transplantation alone, possibly through modulation of proteostasis and increased neuronal differentiation, highlighting the functional superiority of the secretome over cellular therapy (Mendes-Pinheiro et al., 2019). Another BM-MSC secretome study corroborated these findings, showing reversal of Parkinson’s disease–related neurobehavioral and biochemical impairments, including oxidative stress and inflammation (Mahendru et al., 2021). In AD, NSC secretome promoted hippocampal neurogenesis, increased BrdU/Nestin^+^ and BrdU/NeuN^+^ cell populations, and activated the Wnt/β-catenin pathway, thereby improving memory performance and reducing neurotoxicity (Hijroudi et al., 2022). MSC-derived EVs reduced Aβ plaque burden in early-stage APP/PS1 mice, likely due to direct enzymatic degradation by neprilysin and immunomodulatory effects (Elia et al., 2019). Additionally, dental pulp stem cell-CM proved more effective than BM-MSC-CM in enhancing hippocampal neurogenesis and cognitive function, suggesting that neural crest–derived MSCs may be superior due to higher expression of neuroprotective and anti-apoptotic factors (Venugopal et al., 2022). Beyond classic neurodegenerative diseases, ASC-derived secretome showed promise in spinal cord injury models. The whole ASC secretome improved motor recovery more effectively than isolated protein or vesicular fractions, underlining the synergistic role of secretome components. Moreover, intravenous delivery outperformed local injections, emphasizing the systemic therapeutic potential of ASC secretome (Pinho et al., 2022). Although cell-based secretomes have shown promise in rejuvenating various tissues across multiple indications, a key challenge in the context of AD lies in identifying and producing the most functionally relevant cell-derived secretome capable of eliciting meaningful therapeutic effects in the aging brain.

## Key Bioactive Circulating Factors

While the therapeutic benefits of young blood, plasma, and secretome-based interventions are increasingly evident in preclinical and clinical studies, elucidating the specific bioactive molecules responsible for these effects is essential for optimizing efficacy, reproducibility, and clinical translation. Importantly, many of these rejuvenation factors act in a pleiotropic manner, convergently modulating multiple hallmarks of aging rather than operating through a single pathway. This interconnected activity underscores their potential to provide broader and more durable benefits in AD, where cognitive decline arises from the cumulative impact of diverse aging-related mechanisms. Here we will cover several key factors with a regenerative potential contributing to modify distinct, yet overlapping, hallmarks of aging. Building on the proteins identified by Kang and Yang (2020), we will provide further information on recent studies covering the therapeutic benefit of these factors, as well as identifying additional key promising factors (**Table 2**).

**Table 2:** Potential of neuroprotective secreted factors modulating Alzheimer’s disease pathophysiology

Factor	AD-associated biological dysfunctions	Mechanism/Effect	Reference
Apelin-13	Mitochondrial dysfunction	Promotes mitochondrial biogenesis, reduces oxidative stress	Kang and Yang, 2020; Chen et al., 2023
	Cellular senescence	Apelin deficiency accelerates aging in mice	Kang and Yang, 2020
	Altered intercellular communication	Reduces neuroinflammation and neuronal apoptosis	Samandari-Bahraseman and Elyasi, 2022; Chen et al., 2023
	Loss of proteostasis	Protects against Aβ-induced neurotoxicity; preserves synaptic proteins	Gazmeh et al., 2022; Lu et al., 2024
GDF-11	Cellular senescence	Suppresses p21 via Smad2 signaling, delays neuronal aging	Wang et al., 2023
	Stem cell exhaustion	Enhances neurogenesis and synaptic density post-injury	Hudobenko et al., 2020
	Vascular aging & BBB dysfunction	Promotes angiogenesis and white matter integrity	Hudobenko et al., 2020
	Altered intercellular communication	Impacts peripheral-brain crosstalk post-exercise	Schon et al., 2023
GDF-15	Loss of proteostasis	Promotes NEP and IDE expression to degrade Aβ	Xiong et al., 2021
	Altered intercellular communication	Reduces inflammation via SIRT1/Nrf2/HO-1 pathway	Xiong et al., 2021
	Neurovascular aging	Associated with NfL, WMHs, and cognitive decline	Chai et al., 2016; Giudici et al., 2023
TIMP-2	Stem cell exhaustion	Enhances hippocampal plasticity and neurogenesis	Ferreira et al., 2023
	Loss of proteostasis	Modulates ECM complexity, enabling synaptic remodeling	Ferreira et al., 2023
	Vascular aging	Altered MMP/TIMP ratio linked to white matter lesions	Kimura et al., 2020; Vervuurt et al., 2023
Klotho	Loss of proteostasis	Enhances autophagy to clear Aβ aggregates	Fung et al., 2022
	Mitochondrial dysfunction	Protects axons and dendrites from amyloid-β toxicity	Shaker et al., 2025
	Cellular senescence	Klotho knockout affects glial/neuronal RNA profiles	Dubnov et al., 2024
	Altered intercellular communication	Suppresses Wnt/β-catenin and NF-κB-mediated inflammation	Li et al., 2022c
	Systemic aging/Organ crosstalk	Modulates kidney–brain axis (renal function–CSF Aβ link)	Ren et al., 2023
Clusterin (CLU)	Loss of proteostasis	Facilitates Aβ clearance, modulates tau pathology	Palihati et al., 2024; Cohn et al., 2025
	Synaptic dysfunction	Restores synaptic proteins, prevents synapse loss	Lish et al., 2025; Zhao et al., 2025
	Neuroinflammation	Suppresses NF-κB and C3 in astrocytes	Lish et al., 2025
	Biomarker potential	Plasma levels correlate with MCI–AD conversion	Tournier et al., 2025
Neprilysin (NEP)	Loss of proteostasis	Degrades soluble and oligomeric Aβ forms	Sekine-Aizawa et al., 2001; Marr et al., 2003
	Epigenetic dysregulation	HDAC inhibition restores NEP expression	Marr et al., 2003
	Modulated by systemic signals	Dopaminergic signaling and irisin upregulate NEP	Watamura et al., 2024; Kim et al., 2025
	AD progression	NEP deficiency accelerates plaque formation	Morito et al., 2025

Aβ: Amyloid-beta (peptide involved in Alzheimer’s disease pathology); AD: Alzheimer’s disease; BBB: blood–brain barrier; C3: complement component 3; CLU: clusterin (a.k.a apolipoprotein J); CSF: cerebrospinal fluid; ECM: extracellular matrix; GDF-11: growth differentiation factor 11; GDF-15: growth differentiation factor 15; HDAC: histone deacetylase; HO-1: heme oxygenase 1; IDE: insulin-degrading enzyme; MCI: mild cognitive impairment; MMP: matrix metalloproteinase; NAD^+^: nicotinamide adenine dinucleotide (oxidized form); NEP: neprilysin; NF-κB: nuclear factor kappa-light-chain-enhancer of activated B cells; NfL: neurofilament light chain; Nrf2: nuclear factor erythroid 2-related factor 2; p21: cyclin-dependent kinase inhibitor 1A; RNA: ribonucleic acid; SIRT1: sirtuin-1; Smad2: mothers against decapentaplegic homolog 2; TIMP-2: tissue inhibitor of metalloproteinases-2; Wnt: wingless-related integration site; WMHs: white matter hyperintensities.

### Apelin-13

Apelin is a bioactive peptide derived from the APLN gene that binds the APJ receptor and is widely expressed in multiple tissues. Its levels decline with age and in stress-induced senescence. Apelin plays protective roles in various diseases, including neurological and metabolic disorders. Notably, it functions as an exercise-induced myokine that improves muscle mass and regenerative capacity in aging by promoting mitochondrial biogenesis and stem cell function (Kang and Yang, 2020). Mice lacking Apelin or its receptor exhibit accelerated aging, while systemic apelin supplementation restores muscle function and healthspan, making it a promising target for muscle rejuvenation in aging (Kang and Yang, 2020).

Apelin-13, has emerged as a promising neuroprotective candidate in AD, showing beneficial effects across multiple experimental paradigms. Clinical observations demonstrate that patients with AD exhibit significantly reduced serum apelin-13 levels, accompanied by decreased total antioxidant status, suggesting a role for apelin deficiency in disease pathogenesis via increased oxidative stress (Yildiz et al., 2021). In preclinical models, intranasal administration of apelin-13 significantly ameliorated cognitive impairments in streptozotocin-induced AD mice, primarily through enhancement of synaptic plasticity and upregulation of the Nrf2-HO-1 antioxidant pathway (Lu et al., 2024). Complementary findings by Chen et al. (2023) demonstrated that apelin-13 also acts through activation of the peroxisome proliferator-activated receptor-gamma coactivator 1-alpha/PPARγ axis, reducing neuronal apoptosis, oxidative stress, and neuroinflammation. Notably, inhibition of peroxisome proliferator-activated receptor-gamma coactivator 1-alpha abolished beneficial effects of apelin-13, underscoring the significance of this signaling pathway. Apelin-13 has also shown efficacy in reversing scopolamine-induced memory deficits and hippocampal neuronal loss in rats. The peptide restored the expression of key synaptic proteins, such as neurexin-1, neuroligin, and postsynaptic density protein 95, thereby promoting synaptic integrity and function (Gazmeh et al., 2022). At the cellular level, apelin-13 protected SH-SY5Y neuroblastoma cells from Aβ-induced neurotoxicity. It significantly reduced reactive oxygen species levels, mitochondrial calcium release, and apoptotic markers (caspase-3 and cytochrome c), highlighting its antioxidant and anti-apoptotic effects (Samandari-Bahraseman and Elyasi, 2022).

Collectively, these findings support apelin-13 as a multi-target agent capable of mitigating cognitive decline and neuronal injury in AD through modulation of oxidative stress, mitochondrial function, inflammation, and synaptic preservation.

### Growth differentiation factor 11 and growth differentiation factor 15

Growth differentiation factor 11 (GDF11), a transforming growth factor beta superfamily member, plays roles in development and tissue homeostasis, including neurogenesis and myogenesis. Initially identified as an anti-aging factor that reverses cardiac and skeletal muscle aging and promotes neurogenesis, a later study has challenged these findings, reporting that GDF11 may impair muscle regeneration, inhibit bone formation, and correlate with frailty (Poggioli et al., 2016). Conflicting results stem from issues in detection methods and protein specificity. Despite ongoing debate, GDF11 remains a significant factor in aging research and potential therapeutic targeting. Recent studies have further expanded our understanding of GDF11 as a modulator of brain aging, neurogenesis, and recovery following injury. Wang et al. (2023) demonstrated that GDF11 is predominantly expressed in excitatory neurons in mice, marmosets, and humans, and its selective deletion in post-mitotic excitatory neurons induces senescence, dendritic pruning, impaired synaptic input, and cognitive decline in mice. Mechanistically, GDF11 suppresses p21 expression via Smad2 signaling, thus acting as a key repressor of neuronal aging and a promoter of cognitive function and lifespan extension (Wang et al., 2023). In the context of brain injury, Hudobenko et al. (2020) reported that systemic GDF11 supplementation in aged mice after ischemic stroke reduced mortality, gliosis, and inflammation while enhancing neurogenesis, angiogenesis, synaptic density, and white matter integrity. These effects translated to improved sensorimotor recovery and reduced brain atrophy, supporting a therapeutic role of GDF11 in post-stroke repair.

Interestingly, Schön et al. (2023) investigated the acute regulation of GDF11 by endurance exercise in healthy young adults. They found that a 90-minute run significantly reduced GDF11 levels in CSF without altering its plasma or serum levels. The extent of reduction was associated with physical fitness and correlated with cognitive function scores, suggesting that GDF11 dynamics may be linked to exercise-related brain changes, although its direct role in mediating neuroplasticity remains to be determined (Schon et al., 2023). However, not all evidence supports GDF11 as a purely beneficial factor. In a study by Yang et al. (2021), higher plasma levels of GDF11 were paradoxically associated with accelerated long-term forgetting in asymptomatic individuals genetically predisposed to familial AD. This association suggests a complex, possibly dose or context dependent role for GDF11 in hippocampal function and memory consolidation in humans (Yang et al., 2021). Together, these findings underscore GDF11 as a multifaceted molecule influencing neuronal senescence, neuroregeneration, cognitive function, and potentially, AD-related cognitive trajectories, warranting deeper investigation into its therapeutic window and context-specific effects.

Growing body of evidence highlights the role of growth differentiation factor 15 (GDF-15) in AD pathophysiology, with studies exploring both its therapeutic and biomarker potential. *In vitro*, bone marrow mesenchymal stem cell-derived exosomes enriched with GDF-15 were shown to significantly alleviate Aβ42-induced injury in SH-SY5Y cells by reducing apoptosis and inflammation while enhancing cell viability. This effect was mediated through activation of the AKT/glycogen synthase kinase-3 beta/β-catenin signaling pathway, promoting the expression of Aβ-degrading enzymes neprilysin (NEP) and insulin-degrading enzyme (Xiong et al., 2021). Conversely, destabilization of GDF-15 mRNA via the upregulation of circLPAR1 promoted oxidative stress and neuroinflammation in APP/PS1 mice, underscoring neuroprotective role of GDF-15 through the SIRT1/Nrf-2/HO-1 axis (Xiong et al., 2021). In support of a systemic role, plasma GDF-15 levels were positively associated with neurodegeneration markers such as neurofilament light chain and progranulin in older adults, independent of *APOE* *ε4* status, further connecting GDF-15 to aging-related neuroinflammation (Giudici et al., 2023). Clinically, CSF GDF-15 levels have been found to inversely correlate with Mini-Mental State Examination scores and positively associate with low Aβ42 levels, suggesting a link between elevated GDF-15 and cognitive decline in early AD (Plantone et al., 2025). Similarly, a Mendelian randomization study confirmed a causal relationship between genetically predicted higher GDF-15 levels and increased AD risk, with no causal effects seen for Parkinson’s disease or ALS, nor reverse causality from AD to GDF-15 (Wu et al., 2021). Another study found GDF-15 to be significantly elevated in AD and cognitively impaired individuals with substantial white matter hyperintensities, independent of cardiovascular comorbidities, suggesting its utility as a marker for vascular contributions to cognitive impairment (Chai et al., 2016). Collectively, these findings position GDF-15 as a promising diagnostic and mechanistic biomarker, with a potential role in both neuroinflammation and neurodegeneration in AD.

### Tissue inhibitors of metalloproteases

Matrix metalloproteinases (MMPs) and their tissue inhibitors (tissue inhibitors of metalloprotease, TIMPs have been linked to age-related neurodegeneration and AD. A growing body of research implicates imbalances in MMPs and TIMPs in AD and related neurodegenerative or cerebrovascular conditions (Rivera, 2019).

Altered ratios of MMPs to TIMPs in CSF, especially decreased MMP-2/TIMP-2 and MMP-14/TIMP-2 were identified in both sporadic and hereditary Cerebral Amyloid Angiopathy, indicating a possible biomarker role and potential contribution to cerebral vascular damage (Vervuurt et al., 2023). Additional studies demonstrated that MMP-2 and TIMP-1/2/3 (a composite of TIMP-1, -2, and -3) levels rise over time in cognitively unimpaired individuals and predict age-related hippocampal and entorhinal cortex atrophy, implicating MMP/TIMP dynamics in normal brain aging (Aksnes et al., 2023). Elevated CSF levels of MMP-2, MMP-3, MMP-10, TIMP-1, and TIMP-2 were also observed in delirium patients, especially following acute trauma such as hip fracture, though low TIMP-4 was uniquely associated with delirium independent of trauma or dementia (Aksnes et al., 2024).

On a mechanistic level, TIMP-2 has emerged as a critical modulator of hippocampal plasticity and extracellular matrix complexity. Ferreira et al. (2023) showed that TIMP-2, abundantly expressed in hippocampal neurons, regulates synaptic remodeling, adult neurogenesis, and extracellular matrix accumulation, processes that deteriorate with age. Loss of TIMP-2 expression impaired hippocampus-dependent memory and increased extracellular matrix density, restricting neuronal migration and plasticity (Ferreira et al., 2023). Similarly, patients with mild cognitive impairment and amyloid-positive positron emission tomography scans showed higher plasma MMP-2, -8, and -9 levels, reduced TIMP-1 and -2, and increased MMP/TIMP ratios in those with white matter lesions, indicating that these imbalances may mediate early microvascular and white matter damage in prodromal AD stages (Kimura et al., 2020). Collectively, these studies establish the MMP/TIMP axis as both a contributor to AD pathogenesis and a promising target for biomarker development and therapeutic intervention.

### Klotho

Klotho, a single-pass transmembrane protein predominantly expressed in the brain and kidney, has emerged as a key regulator of multiple metabolic and neuroprotective pathways relevant to aging and neurodegeneration. Emerging studies have demonstrated that the upregulation of Klotho expression ameliorates cognitive impairments in AD mouse models. Mechanistically, Klotho contributes to neuroprotection by enhancing the autophagy-lysosomal pathway, thereby promoting the clearance of Aβ and other neurotoxic protein aggregates. This regulatory role in autophagy suggests that Klotho may exert therapeutic effects in AD through both modulation of protein homeostasis and reduction of pathological burden, for review (Fung et al., 2022).

Elevated levels of circulating Klotho, particularly among carriers of the KL-VS heterozygous variant, have been shown to be associated with reduced risk factors for AD. Gaitán et al. (2022) showed that Klotho concentrations were higher in CSF than in serum and were elevated in individuals with KL-VS heterozygosity, with additional modulation by sex and age. These findings suggest that both genetic and demographic factors must be considered when interpreting circulating Klotho levels in relation to AD risk. Similarly, Chen et al. (2023) showed that KL-VS heterozygosity slowed cognitive decline in *APOE ε4* non-carriers, emphasizing a genotype-dependent neuroprotective interaction (Chen et al., 2023). Moreover, in a Mendelian randomization study using NHANES data, higher serum Klotho levels were associated with better cognitive function, although no direct causal relationship with dementia was identified (Wu et al., 2023).

Mechanistically, Klotho appears to counteract AD pathology via several avenues. Shaker et al. (2025) found that inducible overexpression of Klotho in human iPSC-derived cortical neurons attenuated Aβ-induced neurotoxicity by preserving axonal and dendritic integrity and reducing apoptosis (Shaker et al., 2025). Complementary to this, Lehrer and Rheinstein (2020) identified structural alignment between specific amino acid sequences of Klotho and Aβ, suggesting a potential physical interaction that may facilitate neuroprotective signaling via FGF21 in the context of amyloid accumulation. Additional insights into systemic interactions were provided by Ren et al. (2023) who highlighted a role for Klotho in the kidney-brain axis, showing that plasma Klotho levels mediated the relationship between renal function and CSF Aβ levels. Finally, evidence from transcriptomic analyses has shown that Klotho knockout disrupts brain RNA profiles linked to aging and AD, particularly affecting neuronal and glial microRNAs and transfer RNA fragments (Dubnov et al., 2024), while also modulating inflammation in AD patient-derived peripheral blood mononuclear cells via the Wnt/β-catenin pathway (Li et al., 2022a). Collectively, these findings establish Klotho as a promising biomolecular target for therapeutic strategies aimed at mitigating AD progression through neuroprotective, anti-inflammatory, and systemic mechanisms.

### Other key promising factors

Clusterin (CLU), a multifunctional glycoprotein, has emerged as a key player in the pathophysiology of AD. Elevated expression of clusterin in AD brain tissue has been closely associated with disease progression, particularly through its involvement in Aβ aggregation and deposition. In addition to modulating Aβ pathology, clusterin influences AD pathogenesis by regulating neuroinflammation, apoptosis, and the clearance of pathological proteins. Given these multifaceted roles, clusterin is increasingly recognized as a potential biomarker and therapeutic target, with further research warranted to clarify its diagnostic and clinical utility in AD (Palihati et al., 2024). Recent findings have elucidated the multifaceted neuroprotective role of clusterin in AD, highlighting its potential as both a biomarker and therapeutic target. Zhao et al. (2025) demonstrated that an AD-protective CLU allele enhances neuronal excitability by promoting neuron-to-astrocyte lipid transfer and lipid droplet formation, altering astrocytic glutamate uptake and neuron–glia communication. Complementarily, Lish et al. (2025) showed that astrocytic CLU suppresses nuclear factor κB-dependent inflammation and C3 secretion; its loss leads to increased microglial phagocytosis, synaptic loss, and elevated tau pathology, linking reduced CLU to exacerbated AD pathology (Lish et al., 2025). Supporting its diagnostic relevance, Tournier et al. (2025) found that CLU levels increase in the AD brain before plasma changes, and plasma CLU elevation correlates with conversion from mild cognitive impairment to AD, making it a promising biomarker. Longitudinal analysis revealed persistent upregulation of CLU expression in the hippocampal CA3 region post-ischemia, suggesting a sustained neuroprotective response in ischemia-linked neurodegeneration (Pluta et al., 2025). Finally, Cohn et al. (2025) identified a brain-penetrant small molecule (DDL-357) that enhances secreted CLU, reduces tau pathology, and improves memory in AD mouse models, offering a viable therapeutic approach. Together, these findings emphasize the central role of CLU in modulating neuroinflammation, synaptic integrity, and cognitive resilience in AD.

NEP, a membrane-bound zinc metallopeptidase, plays a critical role in the degradation of Aβ peptides, particularly Aβ_42_, which is central to the pathogenesis of AD. Studies have shown that NEP expression and activity are significantly reduced in aging and AD brains, especially in the hippocampus and cortex, contributing to Aβ accumulation and plaque formation (Sekine-Aizawa et al., 2001). Experimental models demonstrate that enhancing NEP expression via gene delivery or pharmacological agents reduces Aβ burden, prevents synaptic loss, and improves cognitive performance in AD mice (Marr et al., 2003). Moreover, epigenetic mechanisms, such as histone deacetylase inhibition, have been found to restore NEP expression, indicating its regulation is tightly linked to age-related transcriptional silencing. Given its capacity to clear both soluble and oligomeric forms of Aβ, NEP is considered a promising therapeutic target for disease-modifying interventions in AD. Recent findings highlight NEP as a central enzyme in the degradation of Aβ, with growing evidence positioning it as a critical therapeutic target in AD. Watamura et al. (2024) demonstrated that dopaminergic signaling, particularly via levodopa or chemogenetic activation of ventral tegmental area neurons, upregulates NEP levels and activity in the prefrontal cortex, leading to reduced Aβ deposition and cognitive improvement in AD model mice —effects shown to be NEP-dependent. In another study, Morito et al. (2025) directly compared the roles of NEP and insulin-degrading enzyme, revealing that NEP deficiency accelerated Aβ plaque formation more significantly than insulin-degrading enzyme deficiency in App(NL-F) mice, and that the AD-associated NEP M8V mutation impairs Aβ degradation via altered protein localization rather than catalytic dysfunction (Morito et al., 2025). Furthermore, Kim et al. (2025) explored the exercise-induced hormone irisin, showing it enhances astrocytic NEP secretion, contributing to Aβ clearance and cognitive benefits in AD models, thus establishing irisin as a potential indirect modulator of NEP activity (Kim et al., 2025). Collectively, these findings emphasize the importance of maintaining NEP function for Aβ clearance and suggest that pharmacological, genetic, and lifestyle interventions targeting NEP regulation may hold promise for preventing or slowing AD progression.

## Conclusions

The convergence of aging biology and AD research has opened new avenues for understanding and treating neurodegeneration at its roots. By reframing the hallmarks of aging into functional clusters, we highlight how interconnected mechanisms such as genomic instability, mitochondrial dysfunction, proteostasis loss, inflammation and regenerative decline contribute synergistically to AD progression. Traditional therapies targeting Aβ and tau, or aimed at symptomatic relief, have yielded modest clinical benefit. In contrast, targeting upstream drivers of brain aging through systemic rejuvenation strategies offers a promising paradigm shift. Injectable biologics, which include young plasma, stem cell-derived secretomes, and their derivatives, demonstrate multi-targeted benefits across preclinical models of AD. Furthermore, key circulating factors, such as Apelin-13, GDF11, GDF15, TIMPs, and Klotho, are emerging as central mediators of these effects, offering mechanistic insight and potential for biomarker development. Continued efforts to refine and identify optimal cell sources, define optimal delivery strategies, and understand the molecular underpinnings of rejuvenation will be essential for translating these approaches into safe and effective disease-modifying therapies for AD. Ultimately, integrating aging-targeted interventions with precision neuromedicine holds the potential to delay, halt, or even reverse neurodegenerative processes in AD.

## Search Strategy

A comprehensive literature search was conducted across PubMed/Medline (NCBI platform), Embase (Elsevier), Web of Science (Clarivate Analytics), Cochrane CENTRAL, searches were targeted towards papers published between 2018 and 2025. However, literature searches highlighted a large body of important articles that were published before these dates that describe age-related changes in Alzheimer patients. Combined search terms were used in the WOS database to compliment PubMed searches. Search terms included combinations of “Alzheimer’s disease” (AND), “aging” (AND), “rejuvenation” (AND), “young blood” (AND), “plasma therapy” AND, “senolytic” (AND), “autophagy” (AND), “epigenetic reprogramming” (AND), “metabolic modulators” (AND), “rejuvenation factors” (AND), “secretome” (AND), “aging hallmarks”, “heterochronic parabiosis” (AND), and “cell therapy” using Boolean operators. Searches were limited to English publications, including both preclinical and clinical studies. ClinicalTrials.gov was searched for interventional clinical studies across all phases and recruitment statuses.

## Data availability statement:

*Not applicable.*
